# Transverse Separation of the Outer Retinal Layer at the Peripapillary in Glaucomatous Myopes

**DOI:** 10.1038/s41598-018-30523-5

**Published:** 2018-08-20

**Authors:** Yong Chan Kim, Ho Sik Hwang, Hae-Young Lopilly Park, Chan Kee Park

**Affiliations:** 1 Department of Ophthalmology, College of medicine, Chuncheon Sacred Heart Hospital, Hallym University, Chuncheon-si, Gangwon-do Republic of Korea; 2 Department of Ophthalmology, College of medicine, Seoul St. Mary’s Hospital, The Catholic University of Korea, Seoul, Republic of Korea

## Abstract

Glaucoma specialists often overlook the outer retinal changes because the glaucomatous optic neuropathy typically involves retinal nerve fiber layer (RNFL). By detailed inspection of the outer retina in myopic eyes, we observed a separation of the inner nuclear layer (INL) from the outer nuclear layer (ONL) at the peripapillary sclera (pp-sclera). Therefore, we conducted a retrospective observation of 108 eyes of 108 Korean subjects with myopia assessed by swept-source optical coherence tomography (SSOCT) and divided into normal and glaucomatous eyes. Mean subject age, refractive error and axial length difference between 2 groups were insignificant, respectively. To quantify the ONL-INL separation, straight-line distance from ONL endpoint to INL endpoint was measured at the center of the optic disc by SSOCT horizontal scan. The glaucomatous group had significantly large ONL-INL separation than the non-glaucomatous group (*p* = 0.027) but had no significant difference in INL – Anterior scleral canal opening (ASCO) separation. The width of ONL-INL separation were associated with β-peripapillary atrophy (β-PPA), degree of horizontal tilt of the optic disc and worse glaucomatous RNFL defect by Pearson’s correlation analysis (all *p* < 0.001, respectively). In conclusion, we demonstrate transverse separation of INL from ONL at the peripapillary region, which was significantly associated with glaucomatous optic nerve damage. These observations may be of interest to elucidate the role of PPA in glaucoma pathogenesis and a clinical index to take notice for myopic subjects.

## Introduction

The optic nerve head (ONH) is a major bottleneck of the retinal ganglion cell axons. Since it is densely packed and simultaneously has to pass through between the lamina cribrosa, it is a major weak point of the visual pathway. Therefore, proper evaluation of the ONH is of high importance for the physiology and pathophysiology of the optic nerve.

Histologic investigations reveal that the ONH is a three-layered hole consisting of the Bruch’s membrane (BM), peripapillary choroid and peripapillary sclera^1–3^. Emmetropic eyes have the three layers gathered straight and well aligned with one another, seen as the peripapillary ring in the conventional ophthalmoscopy^4^. However, the axially myopic eyes shows markedly elongated and thinned peripapillary scleral flange which distorts its even alignment^5^. Consequently, the multilayered peripapillary ring is slanted towards the temporal, resulting in the separation and exposure of each layers. The amount of distortion in the peripapillary are individually varied which requires specific indicators to assess the peripapillary alteration properly.

The elongated peripapillary region has been defined as two separate regions into the beta zone peripapillary atrophy (β-PPA) and the gamma zone PPA (γ-PPA) with respect to the BM^6,7^. Clinical observational studies regarding the PPA persistently suggest that the β-PPA was associated with glaucoma, while γ-PPA was associated with axial myopia^8–10^. There has been efforts to explain glaucoma development with increasing β-PPA but these explanations seems inadequate^11–13^.

During routine clinical practice, glaucoma specialists often overlook the outer retinal changes because the glaucomatous optic neuropathy typically involves retinal nerve fiber layer (RNFL) and retinal ganglion cell layer (GCL) thinning in the inner retinal layer^14,15^. Although detecting early phase of circumpapillary RNFL deterioration is the gold standard in glaucoma diagnosis and management, assessing the integrity of the retina as a whole unit should not be overlooked as well. By detailed inspection of the outer retina in myopic eyes, we observed a separation of the inner nuclear layer (INL) from the outer nuclear layer (ONL) at the parapapillary region. This separation observed in the PPA region of the myopes seemed to be related with glaucomatous damage and enlarged with accompanying enlargement of β-PPA. On the basis of this observation, we hypothesized that separation of the two retinal nuclear layers may interrupt the integrity of the peripapillary retina, leading to further optic nerve damage. We retrospectively collected clinical data from the glaucomatous myopic subjects who had horizontal scan of the optic nerve head with SSOCT and classified into two groups with respect to the separation width of INL from ONL. The purpose of the present study was to describe the outer retinal separation in the peripapillary of glaucomatous myopes and to investigate factors associated with such changes.

## Materials and Methods

This investigation was a retrospective observational study of 108 subjects who visited the glaucoma clinic of Seoul Saint Mary’s Hospital between September 2016 and November 2017. Informed consent for study participation was obtained. The study was approved by the Seoul St. Mary’s Hospital Institutional Review Board. It followed the tenets of the Declaration of Helsinki.

Each subject received an comprehensive eye examination including measurement of best-corrected visual acuity (BCVA), refraction, slit-lamp biomicroscopy, gonioscopy, Goldmann applanation tonometry, standard automated perimetry (Humphrey Visual Field Analyzer; 24-2 Swedish Interactive Threshold Algorithm; Carl Zeiss Meditec, Inc., Dublin, CA, USA), central corneal thickness by ultrasound pachymetry (Tomey Corporation, Nagoya, Japan), axial length with ocular biometry (IOL Master; Carl Zeiss Meditec, Inc.) and a review of their medical history. Automated RNFL thickness measurements were generated along a standard 3.4 mm circle centered on the optic disc using SSOCT (DRIOCT Triton, Topcon Corporation, Tokyo, Japan).

To be included in the present study, subjects were required to have myopia with axial length longer than 24.0 mm and to have an apparent temporal PPA on horizontal OCT scan image with a width of 200 µm or more measured by the built-in caliper tool of the SSOCT. NTG was defined as having glaucomatous optic neuropathy, such as rim thinning, notching, RNFL defect, glaucomatous visual field defect, an open iridocorneal angle and by the absence of a history of elevated intraocular pressure (IOP) >21 mm Hg. Glaucomatous visual field defect was defined as (1) outside normal limits on glaucoma hemifield tests; or (2) 3 abnormal points, with a P < 5% probability of being normal, 1 abnormal points with P < 1% probability of being normal by pattern deviation; or (3) pattern standard deviation of 5% confirmed on 2 consecutive reliable tests (fixation loss rate <20%; false-positive and false-negative error rates <25%). The exclusion criteria were: (1) history or evidence of other optic neuropathies or congenital anomalies of the optic disc; (2) signs of pathologic myopia including myopic choroidal neovascularization, lacquer crack, angioid streak; (3) extremely myopic eyes with an axial length >30 mm; and (4) eyes with poor image quality in which the PPA was not delineated clearly on OCT. Eligibility was determined by 2 glaucoma specialists (Y.C.K. and H.L.P.), who evaluated the optic disc appearance on stereoscopic disc photographs, RNFL defects on red-free fundus photographs, and results of VF examinations. Evaluators were masked to all other patient and ocular data, and an eye was excluded from study analyses if a consensus could not be reached. When both eyes were eligible, one eye was chosen randomly per subject for data analysis.

Tomographic images of the peripapillary fundus were taken by using the SSOCT. The detailed specifications of the SSOCT have been described^16^. Briefly, a 3D imaging data set was acquired for each subject with a raster scan protocol of 512 × 256 A-scans per data set. Each 3D scan covered an area of 12 mm × 9 mm which was enough to cover the whole peripapillary region^17^. Each horizontal line scan was scanned 27 µm apart, vertically. DRIOCT triton software provides a measurement tool to draw straight lines. Two observers (YCK and HYLP) that were masked to all other ocular and patient information manually measured two parameters on the raster image. (1) γ-PPA, which was measured from the anterior scleral canal opening (ASCO) to BM endpoint, and (2) β-PPA, which was measured between BM endpoint to the beginning of the retinal pigment epithelium (RPE) with underlying BM at the center of the optic disc^6^.

In addition to measurements of the PPA, optic disc tilt measurements were identified by two different measure, horizontal disc tilt, and vertical disc tilt, respectively. Horizontal and vertical tilt angle was measured using the clinical disc margin as the ONH plane and the imaginary line connecting each BM margin as the reference plane^18^. Degree-of-tilt was defined as the angle between the reference plane and the ONH plane. Angle measurements were performed by two observers (YCK and HYLP) with the software intrinsic angle tool. A positive degree of horizontal tilt indicated tilt towards temporal, and a negative horizontal tilt indicated tilt towards nasal. A positive degree of vertical tilt indicated tilt towards inferior, and a negative vertical tilt indicated tilt towards superior.

**Figure 1:**
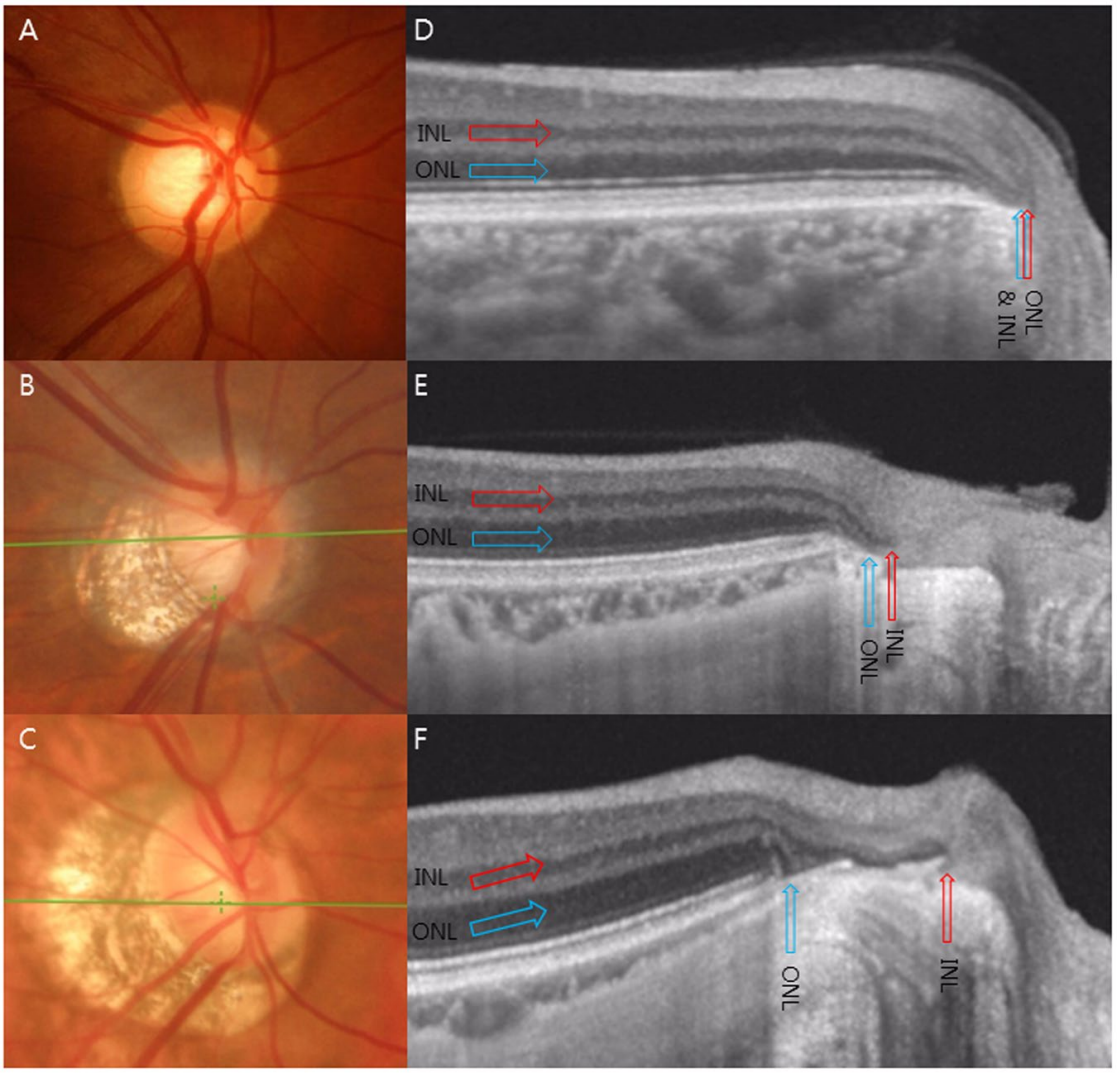
Representation of transverse separation of the inner nuclear layer (INL) endpoint from the outer nuclear layer (ONL) endpoint. The INL of the retina (Red arrows) and the ONL of the retina (Blue arrows) assembled and ended at the BM in the emmetropic eye (**A**,**D**). The elongated peripapillary region of the myopes distorts this configuration, which resulted in various endpoints of each layer (**E**,**F**).

**Figure 2:**
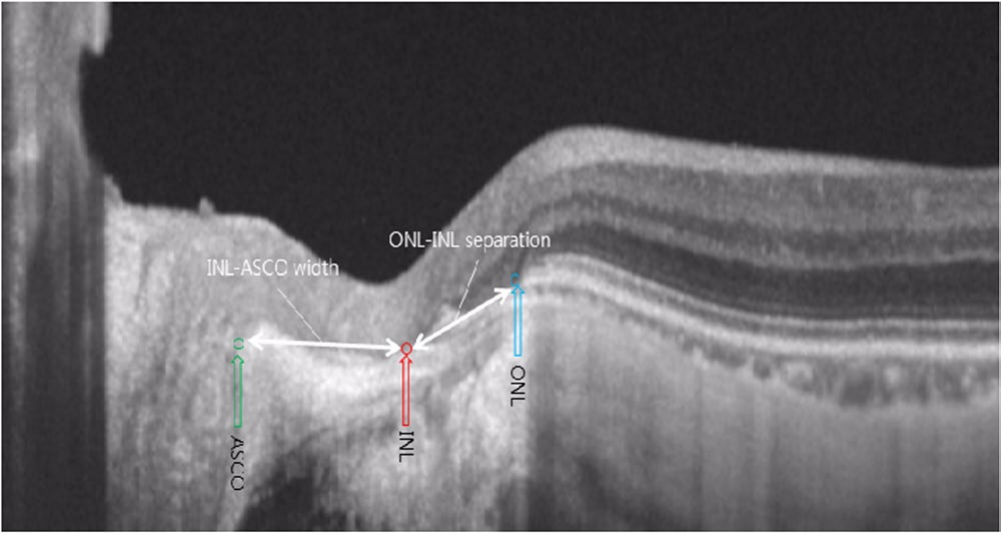
Measurement of the main outcome measures. ONL-INL separation was defined as the straight-line distance from the ONL to INL endpoint. The INL-ASCO width was measured as the straight-line distance from the INL endpoint to ASCO.

The parameters of the outer retinal layer separation were measured at the center of the optic disc using the same section as above. Advances in OCT enable to discriminate the individual retinal layers with high-resolution noninvasive real-time imaging^19^. In the OCT images, the INL of the retina is defined as a relatively hyporeflective zone, internal to hyperreflective outer plexiform layer^20^. The ONL is defined as the hyporeflective zone between the external limiting membrane and the outer plexiform layer^20^. These individual retinal layers assemble and ends at the BM (Fig. 1D)^21^. However, elongated peripapillary region of myopes distorts this configuration, resulting in various endpoints of each layer (Fig. 1E,F). The endpoint of the ONL was defined as the point where the continuous hyporeflective ONL merges at the surface of the BM or sclera. The endpoint of the INL was defined as the point where the continuous hyporeflective INL merges at the surface of BM or RPE. ONL-INL separation was defined as the straight-line distance from ONL endpoint to INL endpoint (Fig. 2). INL-ASCO width was measured as the straight-line distance from INL endpoint to ASCO (Fig. 2). For the sub-analysis, each observer, who was masked with the clinical information of the OCT image, classified each eye into 2 categories with respect to amount of ONL-INL separation: (1) Separated group (ONL-INL separation ≥ 200 µm) and (2) Non-separated group (ONL-INL separation <200 µm).

### Statistical Analysis

Interobserver reproducibility in measurement of the ONL-INL separation and INL-ASCO width were evaluated by calculating intraclass correlation coefficients^22^. Comparison between 2 groups was performed with the chi-square and Student’s t tests. To identify the associated factors with ONL-INL separation, Pearson’s correlation analyses were used. P < 0.05 was considered to be statistically significant.

## Results

A total 149 eyes of 149 subjects who had more than 1 year of follow-up using OCT were included in the study. Of the initial subjects, 9 eyes were excluded because of a history or evidence of non-glaucomatous optic neuropathy (4) or juvenile glaucoma (5). Of the remaining 140 eyes, 32 eyes with minimal PPA (<200 µm) were excluded, leaving a final sample of 108 eyes of 108 subjects.

The mean subject age and refractive error were 44.34 ± 11.96 years (range, 14–72 years) and −4.57 ± 3.16 diopters (range, −10.75 to + 1.0 diopters), respectively. 41 eyes were assigned as the non-glaucomatous group and 67 were assigned as the glaucomatous group.

Table 1 summarizes the comparison of baseline characteristics between the non-glaucomatous group and glaucomatous group. No significant differences were found between groups with regard to age, refractive error, axial length and central corneal thickness. Mean deviation and pattern standard deviation of visual field were more glaucomatous in the non-glaucomatous group and glaucomatous group (all P < 0.001, respectively).

**Table 1:** Demographics and Clinical Characteristics.

	Non-glaucomatous Myopes (n = 41)	Glaucomatous Myopes (n = 67)	P^†^
Age, years*	41.33 ± 12.27	45.71 ± 11.23	0.082
Spherical equivalent, diopter*	−4.29 ± 2.78	−4.84 ± 3.29	0.124
Axial length, mm*	26.26 ± 1.09	26.53 ± 1.21	0.321
CCT, μm*	538.92 ± 55.36	518.98 ± 50.83	0.111
Visual field MD, dB*	−1.19 ± 1.58	−7.17 ± 5.51	**<0**.**001**^**‡**^
Visual field PSD, dB*	1.75 ± 0.79	7.86 ± 4.41	**<0**.**001**^**‡**^
Average RNFL thickness, μm*	96.44 ± 11.18	74.29 ± 13.01	**<0**.**001**^**‡**^

CCT: central corneal thickness; MD: mean deviation of perimetry; PSD: pattern standard deviation of perimetry; RNFL: retinal nerve fiber layer.

^*^Data are presented as mean ± standard deviation unless otherwise indicated.

^†^independent t-test for continuous variables.

^‡^Statistically significant values (P < 0.05) are shown in bold.

^§^χ² test for categorical variables.

Table 2 shows the amount of outer retinal layer separation and peripapillary region characteristics of the two groups. There was excellent interobserver reproducibility in measurement of the ONL-INL separation width, INL-ASCO width, β-PPA width, γ-PPA width, horizontal tilt and vertical tilt (intraclass correlation coefficient = 0.998, 0.972, 0.842, 0.882, 0.996 and 0.987, respectively). INL-ASCO separation, γ-PPA width and total PPA width was similar between the two groups (P = 0.741, P = 0.534 and P = 0.381). However, ONL-INL separation and β-PPA width was significantly larger in the glaucomatous group (P = 0.006 and P = 0.027, respectively).

**Table 2:** Comparison of the Outer Retinal Layer Separation and Peripapillary Region Characteristics between the 2 groups.

	Non-glaucomatous Myopes (n = 41)	Glaucomatous Myopes (n = 67)	P^†^
ONL-INL separation, μm*	212.51 ± 169.26	329.78 ± 180.91	**0**.**006**^**‡**^
INL –ASCO separation, μm*	341.28 ± 149.91	318.44 ± 138.22	0.741
β-PPA width, μm*	214.74 ± 172.24	316.11 ± 200.75	**0**.**027**^**‡**^
γ-PPA width, μm*	357.18 ± 226.34	329.74 ± 220.30	0.534
Total PPA width, μm *	569.42 ± 153.82	631.51 ± 179.42	0.381
Horizontal tilt, degrees*	10.29 ± 6.62	17.36 ± 6.31	**<0**.**001**^**‡**^
Vertical tilt, degrees*	1.37 ± 2.84	6.31 ± 9.19	**0**.**008**^**‡**^

ONL: outer nuclear layer; INL: inner nuclear layer; β-PPA: beta zone peripapillary atophy; γ-PPA: gamma zone peripapillary atophy.

*Data are presented as mean ± standard deviation unless otherwise indicated.

^†^Independent t-test for continuous variables.

^‡^Statistically significant values (P < 0.05) are shown in bold.

Table 3 compares the non-separated group (separation under 200 µm) and separated group (separation over 200 µm). There were no significant differences in the baseline characteristics but the separated group had worse glaucomatous indications in visual fields and RNFL thickness.

**Table 3:** Demographics and Clinical Characteristics of the non-separated group (separation under 200 µm) and separated group (separation over 200 µm).

	Non-Separated ONL-INL group (n = 52)	Separated ONL-INL group (n = 56)	P^†^
Age, years*	42.03 ± 12.57	44.77 ± 9.58	0.317
Spherical equivalent, diopter*	−4.40 ± 2.82	−5.39 ± 3.38	0.210
Axial length, mm*	26.39 ± 1.15	26.63 ± 1.00	0.374
CCT, μm*	523.78 ± 56.15	527.37 ± 50.57	0.764
Visual field MD, dB*	−3.92 ± 3.05	−5.29 ± 3.08	**0**.**003**^**‡**^
Visual field PSD, dB*	4.11 ± 4.09	7.33 ± 4.60	**0**.**002**^**‡**^
Average RNFL thickness, μm*	90.72 ± 13.64	74.06 ± 14.26	** < 0**.**001**^**‡**^

CCT: central corneal thickness; MD: mean deviation of perimetry; PSD: pattern standard deviation of perimetry; RNFL: retinal nerve fiber layer.

*Data are presented as mean ± standard deviation unless otherwise indicated.

^†^Independent t-test for continuous variables.

^‡^Statistically significant values (P < 0.05) are shown in bold.

^§^χ² test for categorical variables.

**Figure 3:**
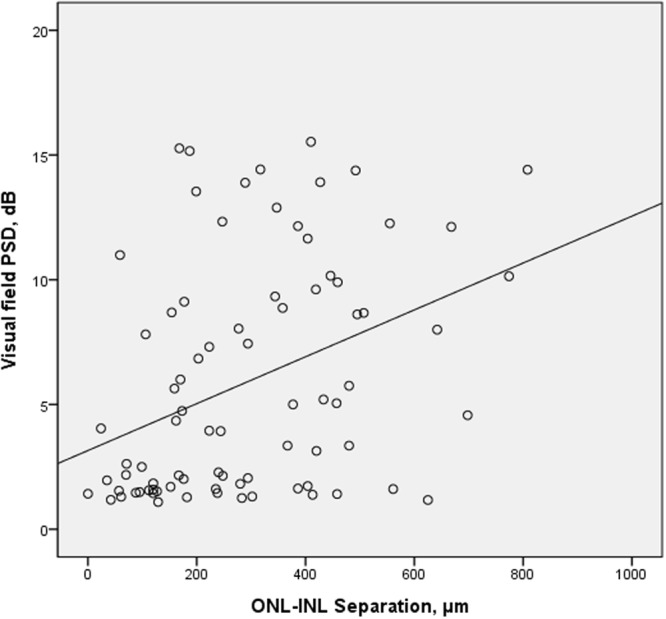
Scatter plot showing the ONL-INL separation and the visual field PSD were significantly correlated (r = 0.375 and P = 0.001).

On correlation analysis, ONL-INL separation was significantly associated with axial length, pattern standard deviation of visual field, β-PPA width and horizontal disc tilt (Fig. 3). INL-ASCO separation was significantly associated with axial length, horizontal disc tilt, γ-PPA width but had no significance with visual field parameters or RNFL thickness (Table 4). Linear regression analysis was done regarding the amount of ONL-INL separation. In the univariate and multivariate analysis, amount of β-PPA width and horizontal tilt degree was associated with ONL-INL separation (Table 5).

**Table 4:** Factors Associated with Outer Retinal Layer Separation.

Variables	ONL-INL Separation		INL to ASCO Separation	
	*R*	P *	*R*	P*
Age, years	0.109	0.330	−0.084	0.454
Spherical equivalent, diopter	**−0**.**271**	**0**.**015**^**†**^	**−0**.**303**	**0**.**006**^**†**^
Axial length, mm	**0**.**340**	**0**.**002**^**†**^	**0**.**266**	**0**.**016**^**†**^
CCT, μm	0.138	0.223	−0.026	0.818
Visual field MD, dB	**−0**.**332**	**0.002** ^**†**^	0.001	0.993
Visual field PSD, dB	**0**.**375**	**0**.**001**^**†**^	0.051	0.653
Average RNFL thickness, μm	**−0**.**455**	**<0**.**001**^**†**^	−0.103	0.359
β-PPA, μm	**0**.**897**	**<0**.**001**^**†**^	0.070	0.531
γ-PPA, μm	0.075	0.501	**0**.**537**	**<0**.**001**^**†**^
Horizontal tilt, degrees	**0**.**449**	**<0**.**001**^**†**^	**0**.**460**	**<0**.**001**^**†**^
Vertical tilt, degrees	0.118	0.290	**0**.**374**	**0**.**001**^**†**^

R: correlation coefficient; ONL: outer nuclear layer; INL: inner nuclear layer; CCT: central corneal thickness; MD: mean deviation of perimetry; PSD: pattern standard deviation of perimetry; RNFL: retinal nerve fiber layer; β-PPA: beta zone peripapillary atophy; γ-PPA: gamma zone peripapillary atophy.

^*^Pearson’s correlation analysis.

^†^Statistically significant values (P < 0.05) are shown in bold.

**Table 5:** Factors Associated with ONL-INL separation.

	Univariate Analyses		Multivariate Analyses*	
	Beta	P Value	Beta (95% CI)	P Value
Age, per year	1.706	0.330		
Axial length, mm	**53**.**645**	**0**.**002**^**†**^		
CCT, μm	0.462	0.223		
β-PPA, μm	**0**.**842**	**<0**.**001**^**†**^	0.799 (0.701–0.896)	**<0**.**001**^**†**^
γ-PPA, μm	0.062	0.501		
Disc torsion, degrees	0.726	0.495		
Disc foveal angle, degrees	−8.789	0.937		
Horizontal tilt, degrees	**11**.**517**	**<0**.**001**^**†**^	3.041 (0.370–5.712)	**0**.**026**^**†**^
Vertical tilt, degrees	2.722	0.290		

CI = confidence interval; ONL: outer nuclear layer; INL: inner nuclear layer; CCT: central corneal thickness; β-PPA: beta zone peripapillary atophy; γ-PPA: gamma zone peripapillary atophy.

^*^Variables with P < 0.1 in univariate analyses were included in multivariate analyses.

^†^Statistically significant values (P < 0.05) are shown in bold.

## Discussion

We describe transversely separated ONL-INL at the peripapillary region which was associated with glaucomatous parameters. The separation is associated with the longer β-PPA width, larger horizontal tilt of the disc, worse glaucomatous visual field and RNFL thickness which are all factors associated with development and progression of glaucoma. As far as we know, this is the first documentation of transversely separated outer retinal layer in the peripapillary region of glaucomatous myopic eye.

Over the years, there have been speculations regarding the pathogenesis of PPA. PPA has been hypothesized as an atrophic change of the RPE-BM complex and subsequent photoreceptor and choriocapillary atrophy^23^. In contrast, it has been regarded as a resultant of scleral stretching associated with development of myopia^24^. Our data clearly demonstrate that in the stretching process of the peripapillary sclera, the retinal configuration can be disorganized and separated transversely. The association of ONL-INL separation with amount of myopia suggests that peripapillary outer retinal separation is the result of scleral stretching associated with axial elongation of the eyeball. The endpoint of each retinal layer merges together at the BM opening of optic disc. As the eyeball grows axially, the temporal peripapillary sclera becomes elongated. In this process, each retinal layer endpoint that sits on the peripapillary sclera is also elongated and eventually results in bilateral separation. The width of ONL-INL separation had significant association with the amount of refractive error and the axial length of the eyeball.

**Figure 4:**
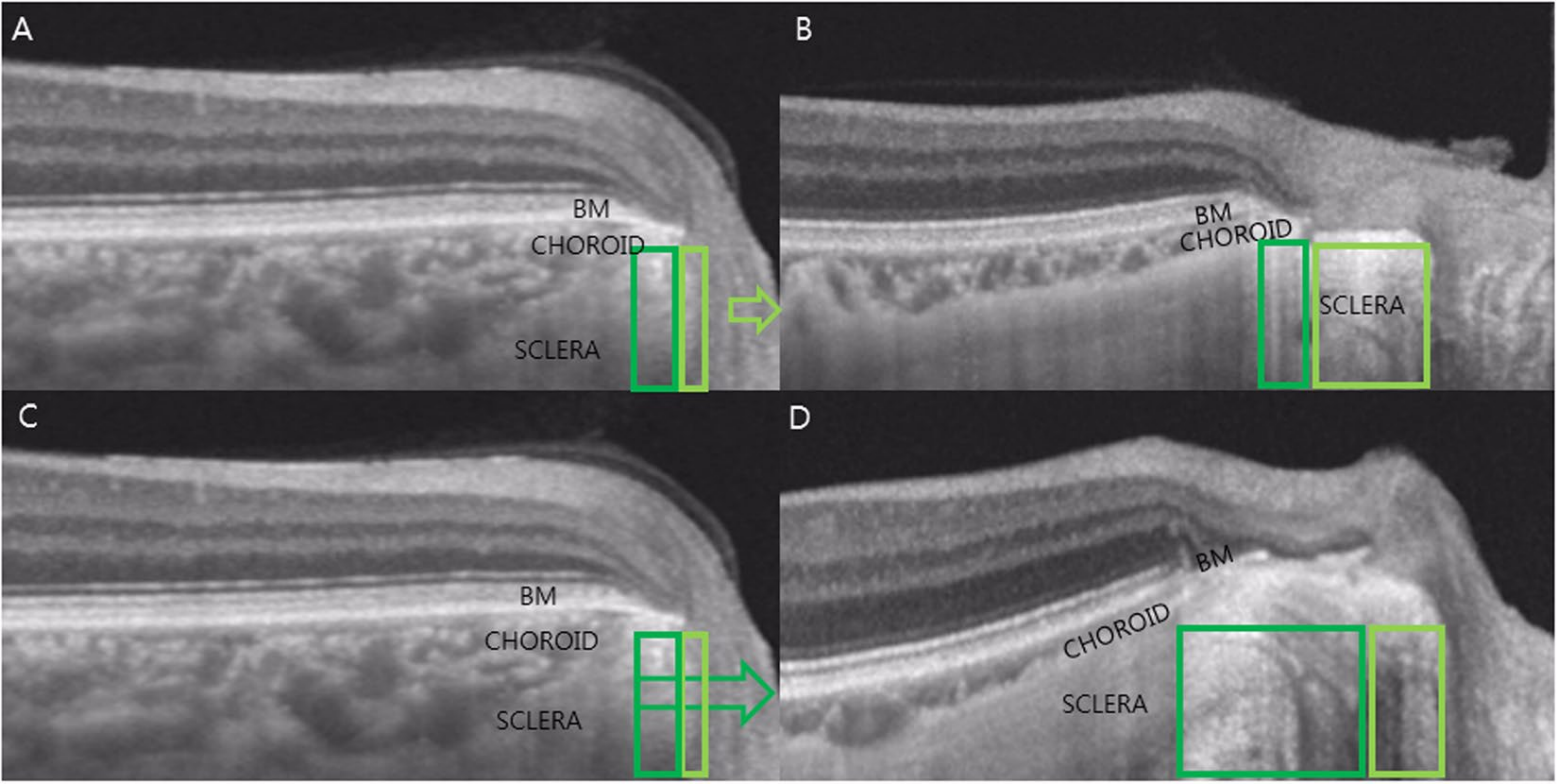
Schematic presentation of the suggested pathogenesis of the uneven alteration at the temporal and nasal sides of the PPA. The emmetropic eye showing no ONL-INL separation (**A**,**C**). With small ONL-INL separations, most of the PPA alterations came from the nasal side of the peripapillary scleral flange (**B**). On the other hand, with large ONL-INL separations, most of the stretching came from the temporal side of the peripapillary scleral flange, which may alter the INL endpoint position that sits above (**D**).

Our observation has implications that the properties of the peripapillary sclera stretching should be taken into consideration as well. So far, PPA has been hypothesized to change equivalently throughout the whole stretched area. However, our data suggest that peripapillary sclera stretches non-uniformly within the elongated scleral flange. As the peripapillary sclera undergoes elongation in myopic eyes, the optic disc margin and the temporal margin of the PPA should be pulled with a same force from the either side (Newton’s third law of motion)^25^. The peripapillary scleral tissue has a characteristic arrangement which transforms from the circumferential orientation adjacent optic disc to the lattice orientation of the posterior pole^26^. Thereby, the temporal and the nasal side of the PPA each undergoes uneven alteration according to the different characteristics and arrangement of each side. This hypothesis is supported by our OCT imaging of the region. In Fig. 4, we compared the OCT images from two subjects, one with minimal ONL-INL separation (Fig. 4B) and the other with large amount of ONL-INL separation (Fig. 4D). With small ONL-INL separation, most of the PPA alterations came from the nasal side of the peripapillary scleral flange. On the other hand, with large ONL-INL separation, most of the stretching came from the temporal side of the peripapillary scleral flange, which may alter the INL endpoint position that sits above. While this finding is not proven longitudinally, this theoretic framework may provide an additional explanation for the association of PPA width with glaucoma^27,28^.

The amount of transverse separation width between ONL-INL was associated with β-PPA width but not with γ-PPA width. Our data shows that the ONL-INL separation had significant association with β-PPA and the INL-ASCO separation had significant association with γ-PPA. In the measurement process, it showed in numerous times that the ONL endpoint matched beginning point of RPE and the INL endpoint matched BM endpoint. Considering the strong association, one hypothesis is that the BM may have some kind of attachment with the INL or the inner retina which moves along with another. However, there is no histologic reference on this speculation and further evaluations should be done.

Manjunath *et al*.^29^ and Lee *et al*.^30^ recently examined the appearances of PPA and retinal morphologic changes with OCT imaging. They reported PPA characteristics as photoreceptor loss and RPE disruption. Additionally, they documented retinal changes such as the RNFL thickness plaque, RNFL cystic spaces and abnormal retinal sloping, but did not examine the changes of individual retinal layer specifically. With detailed investigation, we describe the transverse separation of the outer retinal layer endpoint that seems to be connected with glaucomatous damage.

The question of how the changes in large ONL-INL separation induce glaucomatous optic nerve damage remains to be addressed. In eyes with large ONL-INL separation, disjointed retinal anatomy may induce axonal stress besides the elevated intraocular pressure and its successive mechanical stress to the retinal ganglion cells in the lamina cribrosa level. The pathogenesis of this finding should be addressed in the near future.

Potential limitations of the present study should be discussed. First, all patients were referred to a glaucoma clinic in a tertiary hospital. Further prospective study is needed in subjects with healthy eyes. Second, we are unable to demonstrate conclusively that there is a relationship between the ONL-INL separation and the degree of glaucomatous damage because of the retrospective study design. Only the association discovered with assumption of causality can be reported. The association between changes in ONL-INL separation, glaucomatous damage, axial length and disc change should be evaluated in future prospective studies. Third, our study included only a selected group of individuals who had temporal PPA larger than 200 µm on horizontal OCT scan image. It has remained unclear whether the observations made in this group of individuals can be transferred to groups of patients with small PPA. Fourth, this finding is observed using a relatively novel SSOCT instrument. Whether this finding could be measured with other instruments should be evaluated in future studies.

In conclusion, myopic eyes may develop transverse separation of ONL-INL, which was associated with worse glaucomatous parameters. These observations may be of interest to elucidate the role of PPA in glaucoma pathogenesis and a clinical index to take notice for myopic subjects.
